# Coincidental Occurrence of Multiple Endocrine Neoplasia Type 1 and Breast Cancer in a Young Saudi Girl: A Case Study and Genetic Analysis

**DOI:** 10.7759/cureus.48313

**Published:** 2023-11-05

**Authors:** Amal Alhefdhi, Reem S Alrajhi, Raghad A Almady, Ali S Alzahrani

**Affiliations:** 1 Breast and Endocrine Surgery, King Faisal Specialist Hospital and Research Centre, Riyadh, SAU; 2 Medicine and Surgery, Alfaisal University College of Medicine, Riyadh, SAU; 3 Medicine, King Faisal Specialist Hospital and Research Centre, Riyadh, SAU

**Keywords:** incidental breast cancer, multiple endocrine neoplasia type 1 (men-1), young-onset breast cancer, saudi arabia, riyadh

## Abstract

Multiple Endocrine Neoplasia Type 1 (MEN-1) is an autosomal dominant familial disorder associated with tumors in both endocrine and non-endocrine organs. It is uncommon for MEN-1 to coincide with breast cancer. We present a case of a 15-year-old Saudi girl who exhibited the classic symptoms of MEN-1 and subsequently developed breast cancer. The patient’s breast cancer was diagnosed using ultrasonography and core biopsy, and she was treated with surgical interventions. Despite these treatments, her cancer progressed to a metastatic stage, and her overall health deteriorated, leading to cardiopulmonary arrest at a young age. Although the simultaneous appearance of MEN-1 and breast cancer in our patient may suggest a potential link, our comprehensive genetic analysis found no relationship between her MEN-1 mutation and the onset of breast cancer. This suggests that, in this case, the two conditions co-occurred by chance. Nonetheless, additional research is needed to explore potential associations between MEN-1 and breast cancer.

## Introduction

Multiple Endocrine Neoplasia type 1 (MEN-1) is a rare autosomal dominant genetic condition that affects three to 20 per 100,000 individuals, with over 95% of affected individuals manifesting symptoms by ages 40 to 50 [1]. The disease, which does not show sex predominance, arises from germline mutations in the MEN1 gene. This gene produces menin, a 610-amino acid protein that suppresses cell overproliferation. Mutations can lead to uncontrolled cell proliferation, resulting in tumors in both endocrine and non-endocrine organs [1,2].

Patients typically develop two or more endocrine tumors in organs, the parathyroid gland, gastrointestinal neuroendocrine tissue, and the anterior pituitary gland [3]. Other tumors may also appear, including thyroid neoplasm, adrenal adenoma, pheochromocytoma, lipoma, malignant melanoma, carcinoid tumor, and facial angiofibroma. The classic clinical picture includes tumors in the anterior pituitary, pancreatic islet cell, and parathyroid [4,5]. Recent animal studies suggest a link between MEN-1 gene mutations and alpha-estrogen receptor regulation, possibly implicating them in breast cancer initiation and progression. Exhibiting that menin is a direct activator of alpha-estrogen receptor function by possibly interacting with the AF-2 domain of alpha-estrogen receptor. However, further studies are needed to establish causality [6,7].

Diagnosing MEN-1 requires high clinical suspicion. A detailed MEN-1 genetic analysis and appropriate imaging techniques are essential to confirm the diagnosis [8]. Treatment options depend on disease severity, including surgery, chemotherapy, radiotherapy, and hormone replacement or suppressive therapy. Prognosis varies, with those developing metastatic disease facing a more severe progression and higher mortality rates. Regular monitoring by a multidisciplinary team is crucial to detect and manage such cases promptly. We present a rare MEN-1 and high-grade breast cancer case in a young female patient with a family history of MEN-1 but no known history of breast cancer. Our genetic analysis found no link between her MEN-1 and breast cancer, suggesting a coincidental occurrence.

## Case presentation

A 15-year-old Saudi female middle school student, a known case of MEN-1 was presented to our facility for screening. In regard to her lifestyle, she had healthy habits; however, this does not reflect on her body mass index (BMI) of 16.7 suggesting she was underweight. Her family history was significant for MEN-1 disorder in her mother and four healthy siblings. During screening, the magnetic resonance imaging (MRI) identified a 4 mm microprolactinoma with elevated prolactin levels. Additionally, she exhibited primary hyperparathyroidism, as evidenced by elevated parathyroid hormone and serum calcium levels. An abdominal MRI revealed a 6 mm pancreatic tail nodule and slightly raised chromogranin A levels.

**Figure 1:**
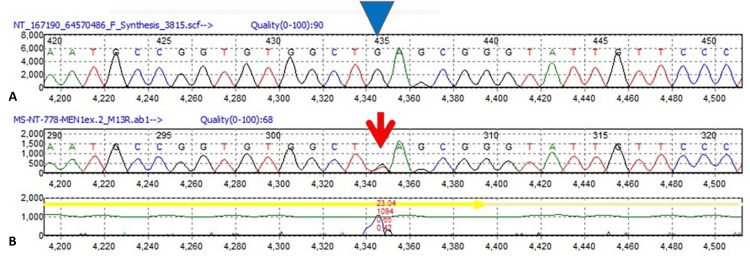
Genetic analysis A) Normal sequence of Exon 2 of the Menin gene. B) The patient’s genomic DNA sequence. A novel heterozygous mutation (c.644 G>T) in Exon 2 of the MEN-1 gene changed G (Arrowhead) to T (arrow) resulting in a stop codon and truncation at codon 222 (p. E222X).

Two years post-screening, she reported a lump in her left breast but had no associated skin changes or nipple discharge. She had no family history of breast or ovarian cancer. Physical examination found a 3 cm x 3 cm lump in the left upper inner breast, while the right breast and both axillae appeared normal. Ultrasound showed an irregular 1.9 cm mass with increased vascularity and scattered calcification in the left breast. Additionally, multiple abnormal nodes with cortices up to 5 mm were detected in the left axilla. A core biopsy confirmed Grade III invasive ductal carcinoma with lymphovascular invasion. Receptor testing showed positive estrogen receptor and progesterone receptors, negative human epidermal growth factor receptor 2, and 42% of cells were positive for Ki-67 protein expression. Fine needle aspiration of the left axillary lymph nodes revealed malignancy. We conducted a genetic analysis using peripheral leukocytes extracted from our patient's blood and her breast tissue. This analysis identified a novel heterozygous mutation (c.644 G>T) in Exon 2 of the MEN-1 gene (Figure 1). Figure 1A displays the normal sequence of Exon 2 of the MEN1 gene, while Figure 1B illustrates the patient's genomic DNA sequence. The identified mutation changes G (indicated by an arrowhead) to T (marked by an arrow), leading to a stop codon and truncation at codon 222 (p. E222X). Therefore, the patient’s breast cancer was likely coincidental and not caused by the MEN-1 mutation.

During the treatment phase, the patient underwent four cycles of epirubicin and cyclophosphamide, then 12 weekly cycles of paclitaxel as part of her neoadjuvant therapy. She then had a wide local excision and an axillary lymph node dissection. The histopathological examination revealed Grade III invasive ductal carcinoma with positive margins. The tumor’s gross examination indicated a size of 40 mm, with satellite foci-the largest being 10 mm. Of the 13 lymph nodes dissected from the axilla, 11 showed metastatic involvement, with some exhibiting extranodal extension.

Subsequently, surgeons performed a left-modified radical mastectomy and inserted a silicone implant. In the postoperative phase, the patient underwent radiotherapy targeting the left chest. She also received anti-estrogen therapy, specifically tamoxifen and goserelin.

At the three-month follow-up, an MRI revealed no residual disease or any malignancy in the contralateral breast, aligning with a breast imaging-reporting and data system (BI-RADs) 2 assessment. Given her elevated parathyroid hormone and calcium levels from before, surgical intervention became necessary. She underwent a total parathyroidectomy, followed by a partial thymectomy, with tissues reimplanted into her right forearm.

However, in October 2017, her tumor markers indicated recurrence, which imaging and biopsy confirmed as metastatic disease. She received hormonal and palliative radiotherapy. By March 2018, liver and bony metastases were evident, and she began treatment with capecitabine. Subsequent pleural fluid cytology tested positive for malignant cells. Despite palliative care, her condition worsened. On January 4th, 2020, aged 23, she was admitted to the King Faisal Specialist Hospital and Research Centre in grave condition. She experienced severe hematemesis during her stay, leading to cardiopulmonary arrest from her advancing malignancy.

## Discussion

MEN-1 is a rare genetic disorder exhibiting autosomal dominant inheritance, following the Mendelian pattern. This condition prompts tumor development in various endocrine and non-endocrine organs, particularly the pituitary gland, pancreatic islet cells, and parathyroid gland. Tumor development is tied to the inactivation of the MEN-1 gene on chromosome 11q13, which typically suppresses tumor formation by regulating cell proliferation [8-10].

Despite its rarity, MEN-1 has a notable mortality rate of approximately two-thirds of its patients. It is crucial to identify and diagnose individuals with MEN-1 early [11]. Patient symptoms can vary widely, from being asymptomatic to presenting invasive carcinoma. The most frequent presentation is hyperparathyroidism, often coupled with hypercalcemia and reduced bone density. Pancreatoduodenal neuroendocrine tumors correlate with higher mortality in MEN-1 patients, while pituitary tumors account for approximately 40% of initial presentations, especially in women [11]. Our patient’s classic MEN-1 symptoms necessitated surgical intervention, including hyperparathyroidism and hypercalcemia.

Several researchers have highlighted atypical MEN-1 presentations, such as the onset of breast cancer. The relationship between MEN-1 gene changes and breast cancer genesis remains ambiguous. Dreijerink et al. posited that women with MEN-1 may have an increased risk of developing breast cancer [12]. Jeong et al. described a case where a previously healthy 45-year-old woman presented MEN-1 symptoms first through a breast mass [13]. Cheah et al. discussed a woman in her thirties with MEN-1 who developed a histologically confirmed ductal breast tumor [14]. Notably, most reported cases in young women were also histologically ductal. These findings raise questions about the need to establish a targeted breast cancer screening for this demographic. Interestingly, in some cases, genetic analyses of resected tumors showed a loss of heterozygosity at the MEN-1 locus, suggesting a possible link between MEN-1 and breast pathology. Our patient’s genetic assessment, which identified a novel heterozygous mutation (c.644 G>T) in Exon 2 of the MEN-1 gene, indicated that her breast cancer was likely an incidental finding and not directly linked to her MEN-1 mutation.

Diagnosing MEN-1 demands a high clinical suspicion, as its detection relies on its characteristic clinical manifestations. The diagnostic process typically involves both genetic analysis and imaging to detect tumor growth [15]. We applied this comprehensive approach to diagnose our patient with MEN-1. Treatment options include surgery, chemotherapy, radiotherapy, and replacement or suppressive therapies. Regular monitoring for tumor development is also essential [16]. Our patient received the recommended treatments, including surgical and palliative care, complemented by regular follow-ups. The prognosis for MEN-1 patients varies and largely depends on their clinical progression. Those with metastatic diseases often face the most significant mortality risk. Our patient’s condition deteriorated following her metastatic disease emergence. Early detection and intervention are pivotal in improving patient survival [17].

## Conclusions

MEN-1 is a familial autosomal dominant inheritance trait caused by germline mutation in the MEN-1 gene. MEN-1 requires a holistic approach with high clinical suspicion and a detailed tumor-targeted workup to diagnose the condition. The treatment of the condition may vary depending on the patient’s case status, as our patient presented with a classical picture of MEN-1, prompting surgical management. Detailed genetic studies indicate that this case of MEN-1 and breast cancer in our patient was coincidental, and no genetic evidence can determine the causality. Thus, further research is required to prove any possible genetic causality between MEN-1 and breast cancer. Establishing such a causality would facilitate the development of early screening programs, potentially enhancing clinical outcomes for this population.
